# Bis(2,2′-bipyridine)(pyridin-2-olato)ruthenium(II) hexa­fluorido­phosphate benzene hemisolvate

**DOI:** 10.1107/S1600536811045454

**Published:** 2011-11-05

**Authors:** Tomohiko Hamaguchi, Isao Ando

**Affiliations:** a Department of Chemistry, Faculty of Science, Fukuoka University, 8-19-1 Nanakuma, Jonan-ku, Fukuoka 814-0180, Japan

## Abstract

In the title compound, [Ru(C_5_H_4_NO)(C_10_H_8_N_2_)_2_]PF_6_·0.5C_6_H_6_, the Ru^2+^ cation has a distorted octa­hedral RuN_5_O coordination environment. This complex is more distorted than the closely related ruthenium complex containing a pyridine-2-thiol­ate ligand [Santra *et al.* (1997 ▶). *J. Chem. Soc. Dalton Trans.* pp. 1387–1393]. The distortion is caused by the difference in size between the O and S atoms. The benzene solvent mol­ecule is situated on a twofold rotation axis.

## Related literature

For the Ru–(pyridine-2-thiol­ate) complex, see: Santra *et al.* (1997 ▶). For similar Ru–(pyridin-2-o­late) complexes, see: Clegg *et al.* (1980 ▶); Cotton & Yokochi (1998 ▶). For an Ru–bipyridine complex, see: Holligan *et al.* (1992 ▶).
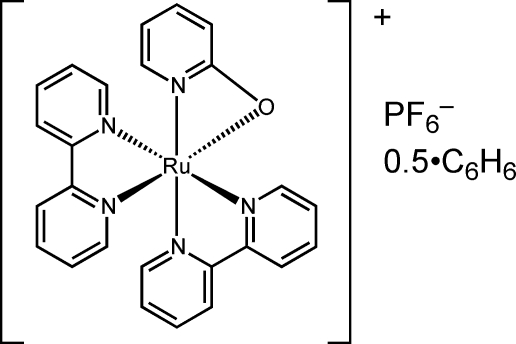


## Experimental

### 

#### Crystal data

[Ru(C_5_H_4_NO)(C_10_H_8_N_2_)_2_]PF_6_·0.5C_6_H_6_
*M* *_r_* = 688.53
Monoclinic, 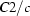

*a* = 21.4180 (4) Å
*b* = 17.5316 (4) Å
*c* = 16.8375 (3) Å
β = 113.3866 (7)°
*V* = 5802.9 (2) Å^3^
*Z* = 8
Mo *K*α radiation
μ = 0.67 mm^−1^
*T* = 200 K
0.26 × 0.22 × 0.10 mm

#### Data collection

Rigaku R-AXIS RAPID diffractometer
Absorption correction: multi-scan (*ABSCOR*; Rigaku, 1995 ▶) *T*_min_ = 0.846, *T*_max_ = 0.937
28444 measured reflections
6649 independent reflections
6068 reflections with *I* > 2σ(*I*)
*R*_int_ = 0.026

#### Refinement

*R*[*F* ^2^ > 2σ(*F* ^2^)] = 0.047
*wR*(*F* ^2^) = 0.155
*S* = 1.14
6649 reflections
364 parameters
3 restraints
H-atom parameters constrained
Δρ_max_ = 2.15 e Å^−3^
Δρ_min_ = −0.52 e Å^−3^

### 

Data collection: *RAPID-AUTO* (Rigaku, 2002 ▶); cell refinement: *RAPID-AUTO*; data reduction: *RAPID-AUTO*; program(s) used to solve structure: *SIR2004* (Burla *et al.*, 2005 ▶); program(s) used to refine structure: *SHELXL97* (Sheldrick, 2008 ▶); molecular graphics: *Yadokari-XG* (Wakita, 2001 ▶; Kabuto *et al.*, 2009 ▶) and *ORTEP-3 for Windows* (Farrugia, 1997 ▶); software used to prepare material for publication: *Yadokari-XG* and *publCIF* (Westrip, 2010 ▶).

## Supplementary Material

Crystal structure: contains datablock(s) I, global. DOI: 10.1107/S1600536811045454/yk2024sup1.cif

Structure factors: contains datablock(s) I. DOI: 10.1107/S1600536811045454/yk2024Isup2.hkl

Additional supplementary materials:  crystallographic information; 3D view; checkCIF report

## Figures and Tables

**Table:** 

Ru1—N2	2.019 (3)
Ru1—N3	2.023 (3)
Ru1—N4	2.050 (3)
Ru1—N5	2.059 (3)
Ru1—N1	2.073 (3)
Ru1—O1	2.146 (3)

**Table:** 

N2—Ru1—N3	79.60 (13)
N2—Ru1—N5	172.74 (12)
N4—Ru1—N5	79.11 (12)
N4—Ru1—N1	165.81 (12)
N3—Ru1—O1	165.07 (11)
N1—Ru1—O1	62.79 (12)

**Fig. 1.**
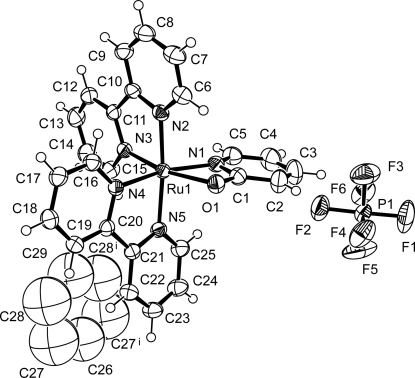
ORTEP drawing for 2 showing 50% probability displacement ellipsoids and arbitrary spheres for H atoms. Symmetry codes: (i) -x + 1, y, -z + 1/2

## supplementary crystallographic information

### Comment

Polypyridine ruthenium complexes have been attracted the interest of researchers for their electrochemistry and photochemistry and also their potential applications as molecular devices. Santra *et al.* (1997) have reported two ruthenium complexes with pyridine-2-thiolate (2-pyS) or pyridin-2-olate (2-pyO). They have revealed the crystal structure of [Ru^II^(bpy)_2_(2-pyS)]ClO_4_ (bpy = 2,2'-bipyridine) complex (1), but X-ray structure study has not been carried out for the 2-pyO complex. Here, we report the crystal structure of [Ru^II^(bpy)_2_(2-pyO)](PF_6_)(C_6_H_6_)_0.5_ (2) and discuss the structural difference between them.

The crystal structure of 2 is shown in Fig. 1. Bond lengths of Ru–O and Ru–N(2-pyO) in 2 are 2.146 (3) and 2.073 (3) Å, respectively. Comparing the bond lengths and that of six-coordinated ruthenium complexes with bidentate 2-pyO derivatives, the Ru–O bond length lies within the range of the reported distances, but Ru–N(2-pyO) is a little shorter than they. For example, in Ru(PPh_3_)_2_(6-methylpyridin-2-olate)_2_ (Clegg *et al.*, (1980), Ru–O and Ru–N(2-pyO) lengths are 2.151 (4) and 2.0919 (5) Å, respectively. In [Ru(PMe_3_)_4_(6-chloropyridin-2-olate)]CF_3_SO_3_ (Cotton *et al.*, (1998), Ru–O and Ru–N(2-pyO) lengths are 2.184 (3) and 2.213 (6) Å, respectively. Average Ru–N(bpy) length in 2 is *ca* 2.04 Å, which is typical length for Ru-bpy complexes (Holligan *et al.*, (1992).

The compound 2 has distorted octahedral geometry on Ru–N_5_O_1_ coordination environment. Coordination polyhedron of 1 is also distorted; however, the distortion of the 2-pyO complex is much more than that of the 2-pyS complex. For example, N1–Ru1–O1 bite angle of 2 is 62.79 (12)° which is steeper than N(2-pyS)–Ru–S(2-pyS) bite angle in 1 (68.6 (2)°). O1–Ru1–N3 and N1–Ru1–N4 angles are 165.07 (11)° and 165.81 (12)°, respectively, that are also steeper than corresponding angles in 1 (167.3 (2)°, 170.1 (3)°) On the other hands, N(bpy)–Ru–N(bpy) angles and Ru–N(bpy) distances in these complexes are almost equal (the average N(bpy)–Ru–N(bpy) bidentate angle: 78.9° (1), 79.36° (2); the *trans*-N(bpy)–Ru–N(bpy) angle: 173.6 (3)° (1), 172.74 (12)° (2); the average Ru–N(bpy) distance: 2.05 Å (1), 2.04 Å (2)) The equality shows that the difference in the degree of distortion is caused by the distinction between the *N,S*- and *N,O*-chelating ligands. Bond lengths of Ru–N(2-pyO) (2.073 (3) Å) and Ru–N(2-pyS) (2.060 (7) Å) are almost same, but the Ru–O bond length (2.146 (3) Å) is shorter than the Ru–S bond length (2.434 (3) Å). Due to the smaller size of O atom with respect to the S atom, the short Ru–O bond would make 2-pyO ligand tilt up to horizontal.

### Experimental

A mixture of [Ru(bpy)_2_Cl_2_].2H_2_O (0.30 g, 0.58 mmol) and 2-hydroxypyridine (0.30 g, 3.2 mmol) in 90 ml ethanol was refluxed. After 1 h, NaOH (0.60 g, 15 mmol) in H_2_O (45 ml) was added, and the mixture was refluxed again for 2 h. After cooling to room temperature, the mixture was concentrated to *ca* 10 ml under reduced pressure. The precipitates were collected by filtration and were washed with a small amount of H_2_O. The precipitates and KPF_6_ (1.12 g) in acetone/methanol (20 ml/20 ml) were evaporated to dryness. 50 ml CHCl_3_ was added to the residium, and the mixture was washed five times with 20 ml H_2_O. The organic layer was dried with Na_2_SO_4_ and was evaporated to dryness. The crude product was recrystallized by vapor diffusion of benzene into an acetone solution at room temperature. Single crystals suitable for single-crystal X-ray analysis were obtained after 1 week.

### Refinement

^1^H NMR spectrum showed that this complex contains a benzene molecule as a crystallization solvent (*δ* = 7.40 ppm in CD_3_CN). The C atoms of benzene molecule (C26–C29) were refined isotropically, and all other non-hydrogen atoms were refined anisotropically. Bond-length restraints of 1.40 (2) Å were applied to all C—C bonds of the benzene, but were not refined except for C27–C28. The H atoms of benzene molecule were not discernible from difference Fourier maps and hence were not included in the final refinement. Other H atoms were placed in calculated positions and were constrained to ride on their parent atoms, with C–H distances of 0.93 Å and with *U*_iso_(H) = 1.2 *U*_eq_(C). The highest residual peak was 0.79 Å from Ru1 atom, and the deepest residual hole was 0.61 Å from C28 atom.

### Crystal data

**Table:**

[Ru(C_5_H_4_NO)(C_10_H_8_N_2_)_2_]PF_6_·0.5C_6_H_6_	*F*(000) = 2752
*M**_r_* = 688.53	*D*_x_ = 1.576 Mg m^−^^3^
Monoclinic, *C*2/*c*	Mo *K*α radiation, λ = 0.71075 Å
Hall symbol: -C 2yc	Cell parameters from 24054 reflections
*a* = 21.4180 (4) Å	θ = 3.0–27.6°
*b* = 17.5316 (4) Å	µ = 0.67 mm^−^^1^
*c* = 16.8375 (3) Å	*T* = 200 K
β = 113.3866 (7)°	Block, black
*V* = 5802.9 (2) Å^3^	0.26 × 0.22 × 0.10 mm
*Z* = 8	

### Data collection

**Table:**

Rigaku R-AXIS RAPID diffractometer	6068 reflections with *I* > 2σ(*I*)
ω scans	*R*_int_ = 0.026
Absorption correction: multi-scan (*ABSCOR*; Rigaku, 1995))	θ_max_ = 27.5°, θ_min_ = 3.0°
*T*_min_ = 0.846, *T*_max_ = 0.937	*h* = −25→27
28444 measured reflections	*k* = −22→22
6649 independent reflections	*l* = −21→21

### Refinement

**Table:**

Refinement on *F*^2^	Primary atom site location: structure-invariant direct methods
Least-squares matrix: full	Secondary atom site location: difference Fourier map
*R*[*F*^2^ > 2σ(*F*^2^)] = 0.047	Hydrogen site location: inferred from neighbouring sites
*wR*(*F*^2^) = 0.155	H-atom parameters constrained
*S* = 1.14	*w* = 1/[σ^2^(*F*_o_^2^) + (0.0839*P*)^2^ + 20.9991*P*] where *P* = (*F*_o_^2^ + 2*F*_c_^2^)/3
6649 reflections	(Δ/σ)_max_ = 0.001
364 parameters	Δρ_max_ = 2.15 e Å^−^^3^
3 restraints	Δρ_min_ = −0.52 e Å^−^^3^

### Special details

**Table:**

Refinement. Refinement of *F*^2^ against ALL reflections. The weighted *R*-factor *wR* and goodness of fit *S* are based on *F*^2^, conventional *R*-factors *R* are based on *F*, with *F* set to zero for negative *F*^2^. The threshold expression of *F*^2^ > σ(*F*^2^) is used only for calculating *R*-factors(gt) *etc*. and is not relevant to the choice of reflections for refinement. *R*-factors based on *F*^2^ are statistically about twice as large as those based on *F*, and *R*- factors based on ALL data will be even larger.

### Fractional atomic coordinates and isotropic or equivalent isotropic displacement parameters (Å^2^)

**Table:**

	*x*	*y*	*z*	*U*_iso_*/*U*_eq_	
Ru1	0.280946 (13)	0.392843 (15)	0.163094 (17)	0.02747 (12)	
O1	0.17546 (13)	0.36260 (16)	0.10031 (17)	0.0372 (6)	
N1	0.22692 (17)	0.38041 (18)	0.2407 (2)	0.0339 (6)	
N2	0.26308 (16)	0.50618 (18)	0.14929 (19)	0.0317 (6)	
N3	0.37222 (15)	0.43350 (17)	0.24506 (19)	0.0302 (6)	
N4	0.31600 (15)	0.38774 (16)	0.06626 (19)	0.0286 (6)	
N5	0.30459 (16)	0.27879 (17)	0.16586 (19)	0.0308 (6)	
C1	0.16864 (19)	0.3599 (2)	0.1738 (3)	0.0363 (8)	
C2	0.1113 (2)	0.3371 (3)	0.1884 (3)	0.0540 (11)	
H1	0.0707	0.3215	0.1420	0.065*	
C3	0.1159 (3)	0.3380 (4)	0.2720 (4)	0.0644 (14)	
H2	0.0776	0.3236	0.2835	0.077*	
C4	0.1753 (3)	0.3597 (3)	0.3397 (3)	0.0601 (13)	
H3	0.1780	0.3600	0.3974	0.072*	
C5	0.2304 (2)	0.3807 (3)	0.3224 (3)	0.0436 (9)	
H4	0.2714	0.3957	0.3685	0.052*	
C6	0.2038 (2)	0.5384 (2)	0.0970 (3)	0.0401 (8)	
H5	0.1649	0.5069	0.0713	0.048*	
C7	0.1978 (3)	0.6152 (2)	0.0796 (3)	0.0458 (10)	
H6	0.1554	0.6359	0.0420	0.055*	
C8	0.2535 (2)	0.6619 (2)	0.1170 (3)	0.0449 (9)	
H7	0.2503	0.7149	0.1043	0.054*	
C9	0.3143 (2)	0.6303 (2)	0.1736 (3)	0.0428 (9)	
H8	0.3531	0.6617	0.2015	0.051*	
C10	0.3179 (2)	0.5522 (2)	0.1889 (2)	0.0330 (7)	
C11	0.37965 (19)	0.5111 (2)	0.2461 (2)	0.0328 (7)	
C12	0.4391 (2)	0.5460 (2)	0.2990 (3)	0.0420 (9)	
H9	0.4437	0.5998	0.2979	0.050*	
C13	0.4921 (2)	0.5020 (3)	0.3539 (3)	0.0491 (10)	
H10	0.5333	0.5254	0.3912	0.059*	
C14	0.4850 (2)	0.4241 (3)	0.3545 (3)	0.0482 (10)	
H11	0.5208	0.3932	0.3925	0.058*	
C15	0.4241 (2)	0.3915 (2)	0.2981 (3)	0.0386 (8)	
H12	0.4194	0.3376	0.2974	0.046*	
C16	0.32495 (19)	0.4469 (2)	0.0210 (2)	0.0350 (7)	
H13	0.3142	0.4967	0.0339	0.042*	
C17	0.3490 (2)	0.4385 (2)	−0.0435 (3)	0.0407 (8)	
H14	0.3551	0.4819	−0.0735	0.049*	
C18	0.3641 (2)	0.3664 (3)	−0.0639 (3)	0.0418 (9)	
H15	0.3798	0.3591	−0.1088	0.050*	
C19	0.35571 (19)	0.3049 (2)	−0.0169 (2)	0.0360 (8)	
H16	0.3661	0.2547	−0.0292	0.043*	
C20	0.33226 (16)	0.3167 (2)	0.0476 (2)	0.0290 (7)	
C21	0.32396 (17)	0.2552 (2)	0.1020 (2)	0.0299 (7)	
C22	0.3350 (2)	0.1789 (2)	0.0904 (2)	0.0364 (8)	
H17	0.3475	0.1635	0.0445	0.044*	
C23	0.3277 (2)	0.1252 (2)	0.1467 (3)	0.0431 (9)	
H18	0.3349	0.0726	0.1398	0.052*	
C24	0.3098 (2)	0.1493 (2)	0.2127 (3)	0.0451 (9)	
H19	0.3053	0.1136	0.2525	0.054*	
C25	0.2985 (2)	0.2257 (2)	0.2202 (2)	0.0382 (8)	
H20	0.2858	0.2417	0.2656	0.046*	
C26	0.5000	0.060 (2)	0.2500	0.261 (14)*	
C27	0.5069 (15)	0.0841 (17)	0.1670 (15)	0.315 (14)*	
C28	0.5073 (13)	0.1660 (16)	0.1687 (14)	0.298 (13)*	
C29	0.5000	0.195 (2)	0.2500	0.280 (15)*	
P1	0.08171 (6)	0.20091 (7)	0.47206 (7)	0.0441 (3)	
F1	0.00943 (19)	0.1814 (3)	0.4681 (3)	0.1085 (16)	
F2	0.15314 (17)	0.2239 (2)	0.4717 (2)	0.0824 (11)	
F3	0.0591 (2)	0.2868 (2)	0.4424 (3)	0.0974 (13)	
F4	0.05521 (19)	0.1762 (2)	0.3729 (2)	0.0841 (11)	
F5	0.1062 (3)	0.11723 (19)	0.5001 (3)	0.1089 (18)	
F6	0.10849 (19)	0.2264 (2)	0.5700 (2)	0.0755 (10)	

### Atomic displacement parameters (Å^2^)

**Table:**

	*U*^11^	*U*^22^	*U*^33^	*U*^12^	*U*^13^	*U*^23^
Ru1	0.02940 (17)	0.02765 (17)	0.02682 (17)	−0.00189 (9)	0.01271 (12)	−0.00315 (9)
O1	0.0341 (13)	0.0431 (15)	0.0339 (13)	−0.0043 (11)	0.0131 (11)	−0.0066 (11)
N1	0.0383 (16)	0.0330 (15)	0.0337 (16)	−0.0018 (12)	0.0177 (14)	−0.0043 (12)
N2	0.0361 (15)	0.0319 (15)	0.0305 (14)	0.0024 (12)	0.0169 (12)	−0.0018 (12)
N3	0.0316 (14)	0.0308 (15)	0.0306 (14)	−0.0012 (11)	0.0149 (12)	−0.0031 (11)
N4	0.0276 (14)	0.0309 (15)	0.0278 (14)	−0.0024 (11)	0.0117 (12)	−0.0032 (11)
N5	0.0342 (15)	0.0284 (14)	0.0311 (14)	−0.0036 (12)	0.0143 (12)	−0.0024 (11)
C1	0.0350 (18)	0.0373 (19)	0.0374 (19)	−0.0013 (15)	0.0153 (16)	−0.0033 (15)
C2	0.040 (2)	0.065 (3)	0.061 (3)	−0.008 (2)	0.024 (2)	−0.002 (2)
C3	0.058 (3)	0.078 (4)	0.077 (4)	−0.003 (3)	0.047 (3)	0.004 (3)
C4	0.075 (3)	0.071 (3)	0.049 (3)	0.003 (3)	0.041 (3)	0.003 (2)
C5	0.053 (2)	0.045 (2)	0.035 (2)	0.0014 (18)	0.0199 (19)	−0.0035 (16)
C6	0.042 (2)	0.040 (2)	0.0370 (19)	0.0065 (16)	0.0151 (17)	0.0008 (16)
C7	0.057 (3)	0.041 (2)	0.042 (2)	0.0138 (18)	0.022 (2)	0.0058 (17)
C8	0.063 (3)	0.033 (2)	0.047 (2)	0.0104 (18)	0.031 (2)	0.0062 (16)
C9	0.058 (2)	0.0318 (19)	0.049 (2)	−0.0037 (17)	0.033 (2)	−0.0042 (17)
C10	0.0435 (19)	0.0289 (17)	0.0350 (18)	−0.0001 (14)	0.0244 (16)	−0.0039 (13)
C11	0.0375 (18)	0.0317 (17)	0.0364 (18)	−0.0061 (14)	0.0221 (15)	−0.0066 (14)
C12	0.043 (2)	0.038 (2)	0.051 (2)	−0.0098 (16)	0.0244 (19)	−0.0139 (17)
C13	0.035 (2)	0.057 (3)	0.052 (2)	−0.0104 (18)	0.0133 (19)	−0.014 (2)
C14	0.035 (2)	0.053 (3)	0.050 (2)	0.0005 (18)	0.0089 (18)	−0.001 (2)
C15	0.036 (2)	0.038 (2)	0.038 (2)	−0.0004 (15)	0.0105 (17)	0.0023 (15)
C16	0.0395 (19)	0.0323 (18)	0.0345 (18)	−0.0007 (14)	0.0162 (15)	−0.0011 (14)
C17	0.051 (2)	0.038 (2)	0.040 (2)	−0.0022 (17)	0.0255 (18)	0.0046 (16)
C18	0.050 (2)	0.044 (2)	0.042 (2)	−0.0015 (18)	0.0287 (19)	−0.0015 (17)
C19	0.0385 (19)	0.0352 (19)	0.0380 (19)	−0.0012 (15)	0.0191 (16)	−0.0058 (15)
C20	0.0253 (15)	0.0320 (17)	0.0279 (16)	−0.0033 (12)	0.0087 (13)	−0.0024 (13)
C21	0.0283 (16)	0.0318 (17)	0.0279 (16)	−0.0021 (13)	0.0092 (13)	−0.0029 (13)
C22	0.0409 (19)	0.0336 (18)	0.0350 (18)	0.0016 (15)	0.0153 (16)	−0.0032 (14)
C23	0.054 (2)	0.0284 (17)	0.047 (2)	0.0025 (17)	0.021 (2)	0.0012 (16)
C24	0.059 (3)	0.034 (2)	0.043 (2)	−0.0004 (18)	0.021 (2)	0.0060 (16)
C25	0.050 (2)	0.0344 (19)	0.0343 (18)	−0.0021 (16)	0.0207 (17)	0.0014 (15)
P1	0.0401 (5)	0.0446 (6)	0.0454 (6)	−0.0092 (4)	0.0148 (5)	−0.0035 (5)
F1	0.063 (2)	0.157 (4)	0.120 (3)	−0.048 (2)	0.051 (2)	−0.029 (3)
F2	0.0651 (19)	0.107 (3)	0.087 (2)	−0.0391 (19)	0.0433 (18)	−0.044 (2)
F3	0.097 (3)	0.055 (2)	0.113 (3)	0.0047 (19)	0.013 (2)	0.006 (2)
F4	0.091 (2)	0.103 (3)	0.0497 (17)	−0.043 (2)	0.0180 (17)	−0.0177 (17)
F5	0.114 (3)	0.0402 (17)	0.110 (3)	−0.0007 (18)	−0.023 (3)	−0.0036 (18)
F6	0.098 (2)	0.082 (2)	0.0528 (17)	−0.0333 (19)	0.0366 (17)	−0.0147 (16)

### Geometric parameters (Å, °)

**Table:**

Ru1—N2	2.019 (3)	C12—C13	1.380 (7)
Ru1—N3	2.023 (3)	C12—H9	0.9500
Ru1—N4	2.050 (3)	C13—C14	1.374 (7)
Ru1—N5	2.059 (3)	C13—H10	0.9500
Ru1—N1	2.073 (3)	C14—C15	1.395 (6)
Ru1—O1	2.146 (3)	C14—H11	0.9500
Ru1—C1	2.549 (4)	C15—H12	0.9500
O1—C1	1.303 (5)	C16—C17	1.383 (5)
N1—C5	1.347 (5)	C16—H13	0.9500
N1—C1	1.357 (5)	C17—C18	1.381 (6)
N2—C6	1.348 (5)	C17—H14	0.9500
N2—C10	1.361 (5)	C18—C19	1.391 (6)
N3—C15	1.337 (5)	C18—H15	0.9500
N3—C11	1.369 (5)	C19—C20	1.382 (5)
N4—C16	1.345 (5)	C19—H16	0.9500
N4—C20	1.364 (4)	C20—C21	1.469 (5)
N5—C25	1.347 (5)	C21—C22	1.387 (5)
N5—C21	1.362 (5)	C22—C23	1.388 (6)
C1—C2	1.403 (6)	C22—H17	0.9500
C2—C3	1.370 (7)	C23—C24	1.378 (6)
C2—H1	0.9500	C23—H18	0.9500
C3—C4	1.383 (8)	C24—C25	1.375 (6)
C3—H2	0.9500	C24—H19	0.9500
C4—C5	1.373 (7)	C25—H20	0.9500
C4—H3	0.9500	C26—C27	1.521 (17)
C5—H4	0.9500	C26—C27^i^	1.521 (17)
C6—C7	1.372 (6)	C27—C28	1.436 (18)
C6—H5	0.9500	C28—C29	1.527 (16)
C7—C8	1.376 (7)	C29—C28^i^	1.527 (16)
C7—H6	0.9500	P1—F1	1.561 (3)
C8—C9	1.388 (7)	P1—F5	1.567 (4)
C8—H7	0.9500	P1—F6	1.581 (3)
C9—C10	1.391 (5)	P1—F2	1.585 (3)
C9—H8	0.9500	P1—F4	1.595 (3)
C10—C11	1.478 (5)	P1—F3	1.599 (4)
C11—C12	1.374 (5)		
			
N2—Ru1—N3	79.60 (13)	N2—C10—C9	121.3 (4)
N2—Ru1—N4	93.66 (11)	N2—C10—C11	113.9 (3)
N3—Ru1—N4	89.89 (11)	C9—C10—C11	124.7 (4)
N2—Ru1—N5	172.74 (12)	N3—C11—C12	121.6 (4)
N3—Ru1—N5	99.46 (12)	N3—C11—C10	114.0 (3)
N4—Ru1—N5	79.11 (12)	C12—C11—C10	124.4 (4)
N2—Ru1—N1	92.77 (12)	C11—C12—C13	119.3 (4)
N3—Ru1—N1	103.71 (12)	C11—C12—H9	120.3
N4—Ru1—N1	165.81 (12)	C13—C12—H9	120.3
N5—Ru1—N1	94.45 (12)	C14—C13—C12	119.8 (4)
N2—Ru1—O1	94.13 (12)	C14—C13—H10	120.1
N3—Ru1—O1	165.07 (11)	C12—C13—H10	120.1
N4—Ru1—O1	104.11 (11)	C13—C14—C15	118.6 (4)
N5—Ru1—O1	88.43 (11)	C13—C14—H11	120.7
N1—Ru1—O1	62.79 (12)	C15—C14—H11	120.7
N2—Ru1—C1	95.17 (12)	N3—C15—C14	122.2 (4)
N3—Ru1—C1	135.66 (12)	N3—C15—H12	118.9
N4—Ru1—C1	134.45 (12)	C14—C15—H12	118.9
N5—Ru1—C1	90.50 (12)	N4—C16—C17	123.0 (4)
N1—Ru1—C1	32.09 (13)	N4—C16—H13	118.5
O1—Ru1—C1	30.73 (11)	C17—C16—H13	118.5
C1—O1—Ru1	92.0 (2)	C18—C17—C16	119.2 (4)
C5—N1—C1	120.6 (4)	C18—C17—H14	120.4
C5—N1—Ru1	145.6 (3)	C16—C17—H14	120.4
C1—N1—Ru1	93.7 (2)	C17—C18—C19	118.3 (4)
C6—N2—C10	118.5 (3)	C17—C18—H15	120.9
C6—N2—Ru1	125.0 (3)	C19—C18—H15	120.9
C10—N2—Ru1	116.1 (2)	C20—C19—C18	120.0 (4)
C15—N3—C11	118.5 (3)	C20—C19—H16	120.0
C15—N3—Ru1	125.7 (3)	C18—C19—H16	120.0
C11—N3—Ru1	115.8 (2)	N4—C20—C19	121.6 (3)
C16—N4—C20	117.8 (3)	N4—C20—C21	114.9 (3)
C16—N4—Ru1	126.6 (2)	C19—C20—C21	123.5 (3)
C20—N4—Ru1	115.5 (2)	N5—C21—C22	121.8 (3)
C25—N5—C21	118.0 (3)	N5—C21—C20	114.8 (3)
C25—N5—Ru1	126.6 (3)	C22—C21—C20	123.4 (3)
C21—N5—Ru1	115.2 (2)	C21—C22—C23	119.1 (4)
O1—C1—N1	111.5 (3)	C21—C22—H17	120.5
O1—C1—C2	127.8 (4)	C23—C22—H17	120.4
N1—C1—C2	120.7 (4)	C24—C23—C22	119.1 (4)
O1—C1—Ru1	57.31 (18)	C24—C23—H18	120.5
N1—C1—Ru1	54.24 (19)	C22—C23—H18	120.5
C2—C1—Ru1	173.4 (3)	C25—C24—C23	119.3 (4)
C3—C2—C1	117.7 (5)	C25—C24—H19	120.4
C3—C2—H1	121.1	C23—C24—H19	120.4
C1—C2—H1	121.1	N5—C25—C24	122.8 (4)
C2—C3—C4	121.2 (4)	N5—C25—H20	118.6
C2—C3—H2	119.4	C24—C25—H20	118.6
C4—C3—H2	119.4	C27—C26—C27^i^	148 (4)
C5—C4—C3	119.0 (5)	C28—C27—C26	105 (3)
C5—C4—H3	120.5	C27—C28—C29	111 (3)
C3—C4—H3	120.5	C28^i^—C29—C28	141 (4)
N1—C5—C4	120.8 (4)	F1—P1—F5	90.9 (3)
N1—C5—H4	119.6	F1—P1—F6	92.8 (2)
C4—C5—H4	119.6	F5—P1—F6	90.6 (2)
N2—C6—C7	122.4 (4)	F1—P1—F2	176.8 (3)
N2—C6—H5	118.8	F5—P1—F2	91.8 (3)
C7—C6—H5	118.8	F6—P1—F2	88.93 (18)
C6—C7—C8	119.7 (4)	F1—P1—F4	87.8 (2)
C6—C7—H6	120.2	F5—P1—F4	89.9 (2)
C8—C7—H6	120.2	F6—P1—F4	179.2 (2)
C7—C8—C9	118.9 (4)	F2—P1—F4	90.47 (19)
C7—C8—H7	120.5	F1—P1—F3	91.2 (3)
C9—C8—H7	120.5	F5—P1—F3	177.8 (3)
C8—C9—C10	119.2 (4)	F6—P1—F3	90.0 (2)
C8—C9—H8	120.4	F2—P1—F3	86.1 (2)
C10—C9—H8	120.4	F4—P1—F3	89.5 (2)
			
N2—Ru1—O1—C1	−93.2 (2)	N5—Ru1—C1—O1	−86.1 (2)
N3—Ru1—O1—C1	−28.7 (6)	N1—Ru1—C1—O1	176.3 (4)
N4—Ru1—O1—C1	172.0 (2)	N2—Ru1—C1—N1	−87.0 (2)
N5—Ru1—O1—C1	93.6 (2)	N3—Ru1—C1—N1	−6.6 (3)
N1—Ru1—O1—C1	−2.2 (2)	N4—Ru1—C1—N1	172.8 (2)
N2—Ru1—N1—C5	−90.5 (5)	N5—Ru1—C1—N1	97.6 (2)
N3—Ru1—N1—C5	−10.5 (5)	O1—Ru1—C1—N1	−176.3 (4)
N4—Ru1—N1—C5	152.6 (5)	O1—C1—C2—C3	179.3 (5)
N5—Ru1—N1—C5	90.4 (5)	N1—C1—C2—C3	1.5 (7)
O1—Ru1—N1—C5	176.3 (5)	C1—C2—C3—C4	−0.9 (8)
C1—Ru1—N1—C5	174.2 (6)	C2—C3—C4—C5	0.2 (9)
N2—Ru1—N1—C1	95.3 (2)	C1—N1—C5—C4	0.6 (6)
N3—Ru1—N1—C1	175.3 (2)	Ru1—N1—C5—C4	−172.7 (4)
N4—Ru1—N1—C1	−21.6 (6)	C3—C4—C5—N1	0.0 (8)
N5—Ru1—N1—C1	−83.8 (2)	C10—N2—C6—C7	2.8 (6)
O1—Ru1—N1—C1	2.1 (2)	Ru1—N2—C6—C7	−169.3 (3)
N3—Ru1—N2—C6	179.9 (3)	N2—C6—C7—C8	−0.7 (6)
N4—Ru1—N2—C6	90.7 (3)	C6—C7—C8—C9	−1.7 (6)
N1—Ru1—N2—C6	−76.7 (3)	C7—C8—C9—C10	1.9 (6)
O1—Ru1—N2—C6	−13.8 (3)	C6—N2—C10—C9	−2.6 (5)
C1—Ru1—N2—C6	−44.6 (3)	Ru1—N2—C10—C9	170.2 (3)
N3—Ru1—N2—C10	7.5 (2)	C6—N2—C10—C11	178.7 (3)
N4—Ru1—N2—C10	−81.7 (3)	Ru1—N2—C10—C11	−8.4 (4)
N1—Ru1—N2—C10	111.0 (3)	C8—C9—C10—N2	0.3 (6)
O1—Ru1—N2—C10	173.9 (2)	C8—C9—C10—C11	178.8 (3)
C1—Ru1—N2—C10	143.1 (2)	C15—N3—C11—C12	1.0 (5)
N2—Ru1—N3—C15	173.5 (3)	Ru1—N3—C11—C12	179.8 (3)
N4—Ru1—N3—C15	−92.7 (3)	C15—N3—C11—C10	−176.5 (3)
N5—Ru1—N3—C15	−13.8 (3)	Ru1—N3—C11—C10	2.3 (4)
N1—Ru1—N3—C15	83.2 (3)	N2—C10—C11—N3	3.9 (4)
O1—Ru1—N3—C15	107.3 (5)	C9—C10—C11—N3	−174.7 (3)
C1—Ru1—N3—C15	86.8 (3)	N2—C10—C11—C12	−173.5 (3)
N2—Ru1—N3—C11	−5.2 (2)	C9—C10—C11—C12	7.9 (6)
N4—Ru1—N3—C11	88.5 (2)	N3—C11—C12—C13	−1.5 (6)
N5—Ru1—N3—C11	167.5 (2)	C10—C11—C12—C13	175.8 (4)
N1—Ru1—N3—C11	−95.6 (2)	C11—C12—C13—C14	0.5 (7)
O1—Ru1—N3—C11	−71.4 (5)	C12—C13—C14—C15	0.8 (7)
C1—Ru1—N3—C11	−92.0 (3)	C11—N3—C15—C14	0.4 (6)
N2—Ru1—N4—C16	4.0 (3)	Ru1—N3—C15—C14	−178.3 (3)
N3—Ru1—N4—C16	−75.5 (3)	C13—C14—C15—N3	−1.3 (7)
N5—Ru1—N4—C16	−175.2 (3)	C20—N4—C16—C17	0.7 (5)
N1—Ru1—N4—C16	120.8 (5)	Ru1—N4—C16—C17	179.9 (3)
O1—Ru1—N4—C16	99.2 (3)	N4—C16—C17—C18	0.7 (6)
C1—Ru1—N4—C16	105.0 (3)	C16—C17—C18—C19	−1.3 (6)
N2—Ru1—N4—C20	−176.7 (2)	C17—C18—C19—C20	0.6 (6)
N3—Ru1—N4—C20	103.7 (2)	C16—N4—C20—C19	−1.5 (5)
N5—Ru1—N4—C20	4.1 (2)	Ru1—N4—C20—C19	179.2 (3)
N1—Ru1—N4—C20	−59.9 (6)	C16—N4—C20—C21	177.5 (3)
O1—Ru1—N4—C20	−81.5 (3)	Ru1—N4—C20—C21	−1.8 (4)
C1—Ru1—N4—C20	−75.8 (3)	C18—C19—C20—N4	0.9 (6)
N3—Ru1—N5—C25	90.8 (3)	C18—C19—C20—C21	−178.1 (4)
N4—Ru1—N5—C25	178.8 (3)	C25—N5—C21—C22	2.2 (5)
N1—Ru1—N5—C25	−14.0 (3)	Ru1—N5—C21—C22	−173.6 (3)
O1—Ru1—N5—C25	−76.5 (3)	C25—N5—C21—C20	−177.6 (3)
C1—Ru1—N5—C25	−45.8 (3)	Ru1—N5—C21—C20	6.6 (4)
N3—Ru1—N5—C21	−93.9 (3)	N4—C20—C21—N5	−3.2 (4)
N4—Ru1—N5—C21	−5.9 (2)	C19—C20—C21—N5	175.8 (3)
N1—Ru1—N5—C21	161.4 (3)	N4—C20—C21—C22	177.0 (3)
O1—Ru1—N5—C21	98.8 (2)	C19—C20—C21—C22	−4.0 (5)
C1—Ru1—N5—C21	129.5 (3)	N5—C21—C22—C23	−1.5 (6)
Ru1—O1—C1—N1	3.2 (3)	C20—C21—C22—C23	178.2 (4)
Ru1—O1—C1—C2	−174.8 (4)	C21—C22—C23—C24	−0.2 (6)
C5—N1—C1—O1	−179.5 (3)	C22—C23—C24—C25	1.2 (7)
Ru1—N1—C1—O1	−3.3 (3)	C21—N5—C25—C24	−1.1 (6)
C5—N1—C1—C2	−1.3 (6)	Ru1—N5—C25—C24	174.1 (3)
Ru1—N1—C1—C2	174.9 (4)	C23—C24—C25—N5	−0.5 (7)
C5—N1—C1—Ru1	−176.2 (4)	C27^i^—C26—C27—C28	0.4 (17)
N2—Ru1—C1—O1	89.4 (2)	C26—C27—C28—C29	−1(3)
N3—Ru1—C1—O1	169.8 (2)	C27—C28—C29—C28^i^	0.4 (18)
N4—Ru1—C1—O1	−10.9 (3)		

Symmetry codes: (i) −*x*+1, *y*, −*z*+1/2.
